# Genome-Wide Identification of Genes Conferring Energy Related Resistance to a Synthetic Antimicrobial Peptide (Bac8c)

**DOI:** 10.1371/journal.pone.0055052

**Published:** 2013-01-31

**Authors:** Eileen C. Spindler, Nanette R. Boyle, Robert E. W. Hancock, Ryan T. Gill

**Affiliations:** 1 Department of Biological and Chemical Engineering, University of Colorado Boulder, Boulder, Colorado, United States of America; 2 Centre for Microbial Diseases and Immunity Research, University of British Columbia, Vancouver, Canada; University of Cambridge, United Kingdom

## Abstract

A fundamental issue in the design and development of antimicrobials is the lack of understanding of complex modes of action and how this complexity affects potential pathways for resistance evolution. Bac8c (RIWVIWRR-NH_2_) is an 8 amino acid antimicrobial peptide (AMP) that has been shown to have enhanced activity against a range of pathogenic Gram-positive and Gram-negative bacteria, as well as yeast. We have previously demonstrated that Bac8c appears to interfere with multiple targets, at least in part through the disruption of cytoplasmic membrane related functions, and that resistance to this peptide does not easily develop using standard laboratory methods. Here, we applied a genomics approach, SCalar Analysis of Library Enrichement (SCALEs), to map the effect of gene overexpression onto Bac8c resistance in parallel for all genes and gene combinations (up to ∼ 10 adjacent genes) in the *E. coli* genome (a total of ∼ 500,000 individual clones were mapped). Our efforts identified an elaborate network of genes for which overexpression leads to low-level resistance to Bac8c (including biofilm formation, multi-drug transporters, etc). This data was analyzed to provide insights into the complex relationships between mechanisms of action and potential routes by which resistance to this synthetic AMP can develop.

## Introduction

A major barrier to the development of antimicrobial peptide-based therapies is the lack of a complete understanding of the complex modes of killing by antimicrobial peptides (AMPs) [1]. Besides the membrane-associated activities of AMPs, a variety of studies have implicated inhibition of DNA, RNA, and protein synthesis, inhibition or specific binding to DNA, inhibition of enzymatic activity, activation of autolysins, inhibition of septum formation and inhibition of cell wall formation as targets of various AMPs [2], [3], [4], [5]. However, it is also likely that AMPs elicit a combination of cell killing strategies [6]. Therefore, direct evidence for specific modes of action has been elusive. The lack of detailed knowledge of the complex mode(s) of action continues to limit our understanding of structure activity relationships, and our ability to take advantage of these compounds [7].

Bac8c (RIWVIWRR-NH_2_) is an 8 amino acid peptide derived through a complete substitution analysis of Bac2A (RLARIVVIRVAR-NH_2_) [8]. It is smaller than bactenecin (also known as bovine dodecapeptide), the smallest known broad spectrum natural antimicrobial peptide, but has enhanced activity against a range of pathogenic Gram-positive and Gram-negative bacteria, as well as yeast [9]. We have previously described efforts to decipher the mode of action of Bac8c [10] and have shown that this AMP appears to interfere with multiple targets, apparently through the disruption of cytoplasmic membrane related functions. Based on the complexity of the Bac8c mode of action, it is not surprising that we were unable to identify mutants with substantial resistance to Bac8c even when we performed comprehensive screening of knockout, over-expression, and chemical mutant libraries (unpublished data). Since this recalcitrance to resistance is a very attractive property for any antimicrobial compound, we sought to improve our understanding of the complex mechanisms employed by this peptide to avoid resistance using a genome-scale library screening method reported previously by our group [11], [12], [13], [14].

The Scalar Analysis of Library Enrichments (SCALEs) approach employs gene-chip technology and precisely designed extra-chromosomal libraries to map the effect of gene over-expression on overall cell fitness (in this case resistance to Bac8c) [13]. Specifically, pooled plasmid-based genomic libraries of different sizes are subjected to selective pressure and the pooled resistant clones quantitatively evaluated by hybridization and quantification of the genomic library DNA inserts using Affymetrix gene-chips and our previously developed SCALEs algorithm. SCALEs has been used previously to identify genes related to increased growth, 3-hydroxypropionic acid tolerance, solvent tolerance, antibiotic tolerance, and anti-metabolite tolerance [11], [13], [14], [15], [16]. The enrichment of particular cloned genes under these circumstances implies that these genes improve fitness in the face of selective pressure and can assist in revealing putative resistance mechanisms that might occur in nature and/or mechanisms of action of the agent used for selection (since overexpression of target sites leads to resistance [17]). Here, we applied this approach to comprehensively characterize potential Bac8c resistance genes, and in turn to develop insights into why resistance to Bac8c does not readily develop and the complex modes of Bac8c action.

## Materials and Methods

### Bacteria, Plasmids, and Materials

*E. coli* strain Mach1-T1^R^ (Invitrogen, Carlsbad, CA.) wild-type W strain (ATCC #9637, S. A. Waksman) Mach1-T1^R^ F^−^ φ80(*lac*Z)ΔM15 Δ*lac*X74 *hsd*R(r_K_^−^m_K_^+^) Δ*rec*A1398 *end*A1 *ton*A containing the pSMART LC KAN empty vector were used for all control studies. Overnight cultures were grown in Luria Bertani (LB) medium. Growth curves were carried out in 3-(N-*morpholino*)propanesulfonic acid (MOPS) minimal medium [18]. For all experiments that required antibiotic to maintain the vector, kanamycin (KAN) was used at 30 µg/ml. Bac8c was synthesized by N-(9-fluorenyl)methoxy carbonyl chemistry from GenScript Corporation (Piscataway, NJ).

### Genomic Library Construction

Drs. Tanya Warnecke and Michael D. Lynch constructed the genomic library, as described previously [13], [16]. Briefly, cultures of the *E*. *coli* K12 were grown overnight in 500 ml of LB at 37°C to an optical density at 600 nm (OD600) of 1. DNA was extracted using a Genomic DNA Purification kit (Qiagen) according to the manufacturer’s instructions. Five samples containing 50 µg of purified genomic DNA were digested using two blunt-end cutting restriction enzymes: AluI and RsaI (Invitrogen). Both enzymes have four base pair recognition sequences and are used in tandem to ensure the random digestion of the genomic DNA. The partially digested DNA was immediately mixed and separated based on size using agarose gel electrophoresis. DNA fragments of 0.5, 1, 2, 4, and greater than 8 kb were excised from the gel and purified with a Gel Extraction Kit (Qiagen).

Ligation of the purified, fragmented DNA with the pSMART LC KAN vectors was performed with the CloneSmart Kit (Lucigen) according to the manufacturer’s instructions. The ligation product was then electroporated into *E*. *coli* (*E*. *Cloni* 10 GF’ Elite Electrocompetent Cells; Lucigen), plated on LB+KAN, and incubated at 37°C for 24 hours. Dilution cultures with one thousandth of the original transformation volume were plated on LB+KAN in triplicate to determine accurate transformation efficiency and to confirm that greater than 10^5^ transformants per library were obtained, corresponding to greater than 99% probability of complete library coverage.

### Transformation of Library DNA

Purified plasmid DNA from each library was introduced into MACH-1™-T1® (Invitrogen) by electroporation. MACH-1™-T1® cultures were made electrocompetent by a standard glycerol wash procedure on ice to a final concentration of 10^11^cells/ml [19]. One thousandth of the volume of the original transformations was plated on LB+KAN agar plates in triplicate to determine transformation efficiency and the adequacy of transformant numbers (>10^6^). The original cultures were combined and diluted to 100 ml with MOPS minimal medium+KAN and incubated at 37°C for 6 hours or until reaching an OD_600_ of 0.20.

### Selection

**Figure 1:**
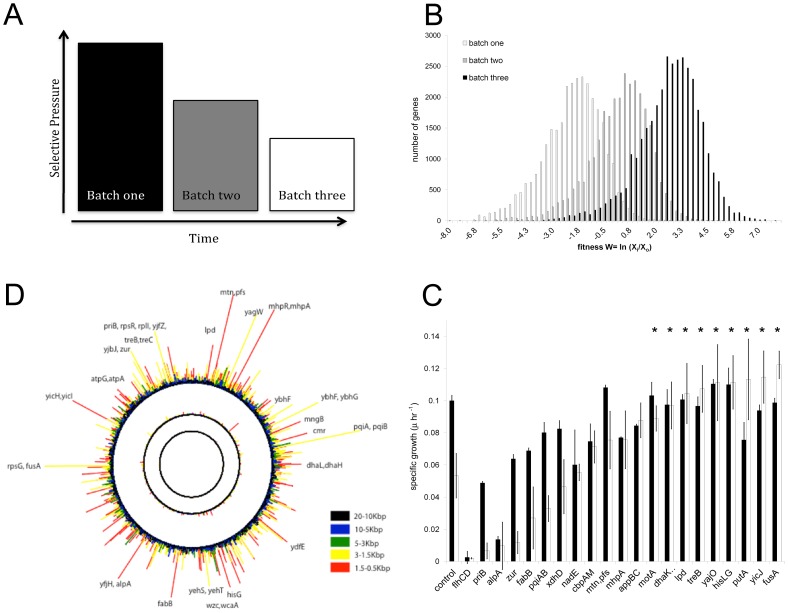
Selection Design. **A)** Strategy: unlike customary selections, our design decreases selective pressure (AMP concentration) through each successive batch. **B)** Histogram of each selected population displaying the increase in relative fitness over time through the three successive batches. **C)** Genome-wide plot of the multi-scale analysis of the fitness of each successive batch culture compared to the control culture at time 0. For each batch, the fitness for each 125-bp position is plotted around the genome for each scale referred to in the legend. A decrease in selective pressure (through each successive batch) moves outwards from the center circle. Circles i, ii, and iii correspond to the (6 µg/ml, 5 µg/ml, 4 µg/ml)/Control, (6 µg/ml, 5 µg/ml)/Control, and 6 µg/ml/Control, respectively. The percentage of the *E. coli* genome is plotted clockwise around the circles. Inserts with high fitness values are labeled. **D)** Growth of SCALEs selected clones. The specific growth of clones grown in 96 well plates and the OD_600_ taken every half hour for eight hours is plotted. Black bars indicate the specific growth of clones in the absence of Bac8c. White bars indicate the specific growth of the clones in the presence of Bac8c at a concentration equal to the IC_50_ of the control (3 µg/ml). The *rpsLG-fusA*, *putA* and *yicJ* clones all had an increased growth rate compared to the control strain in the presence of Bac8c at the IC_50_. *p-value<0.01 is for the white bars (IC50), t-test of control verses clones with resistance.

The MIC of Bac8c for *E. coli* was determined to be 3 µg/ml while the MBC was 6 µg/ml [10]. A repeated batch selection was designed based on work performed previously in the lab [16]. The selection involved decreasing the level of selective pressure (AMP concentrations between the MBC and the MIC) with each batch (Fig. 1a). The newly transformed library was first diluted to an OD_600_ of 0.1. An aliquot of cells (1 ml of 10^7^ cells/ml) was plated at time zero. Two separate selections were preformed one starting at a Bac8c concentration of 7 µg/ml (above the control MBC), and another starting at (6 µg/ml, the control MBC). The first selection (batch one) continued until cells reached mid log (OD_600_ = 0.5), which required 12 h for both AMP concentrations. An aliquot of these cells was diluted and plated on LB+KAN plates (10,000 colonies per plate), and the cultures were then diluted to an OD_600_ of 0.1 and supplemented with 6 µg/ml and 5 µg/ml Bac8c respectively added to the separate selections (batch two). This batch was again grown to mid-log phase, which in this round required 6 h of growth. An aliquot of these cells was then plated and the cultures were again diluted and supplemented with 5 and 4 µg/ml Bac8c respectively to the separate selections. Selected populations were plated onto LB+KAN plates, and colonies were harvested after 24 h by gently scraping the plates into TB medium. The cultures were immediately resuspended by vortexing, and aliquoted into 15×1 mL freezerstock cultures with a final glycerol concentration of 15% (v/v) [20]. The remainder of the culture was pelleted by centrifugation for 15 min at 3000 rpm. Plasmid DNA was extracted according to the manufacturer’s instructions using a HiSpeed Plasmid Midi Kit (Qiagen). To confirm insert sizes and numbers of positive transformants, plasmids were isolated from random clones for each sized library using Qiaprep Spin MiniPrep Kit (Qiagen). Purified plasmids were then analyzed by either PCR using primers SL1 (5′-CAGTCCAGTTACGCTGGAGTC-3′) and SR2 (5′-GGTCAGGTATGATTTAAATGGTCAGT) or by restriction digestion.

### DNA Microarrays

For each array, 3 µg of sample plasmid DNA was mixed with the following control plasmid DNA: 1000 ng pGIBS-DAP (ATCC#87486), 100 ng pGIBS-THR (ATCC# 87484), 10 ng pGIBS-TRP (ATCC# 87485) and 1 ng pGIBS-PHE (ATCC# 87483). The plasmid mixture was digested at 37°C overnight with 1 unit each of AluI and RsaI (Invitrogen) in a reaction containing 50 mM Tris-HCl (pH 8.0), and 10 mM MgCl2. Reactions were heat inactivated at 70°C for 15 min. 10X One Phor All Buffer (Amersham Pharmacia Biotech, Piscataway, NJ) was added to the digestions to a final 1X concentration. 1 µL RQDNAse I (Fisher) was added to the reactions and incubated at 37°C for 2 min followed by heat inactivation at 98°C for 20 min.

One µl of Exonuclease III (Fisher) was added to the reactions and incubated at 37°C for 15 min followed by heat inactivation at 98°C for 20 min. The resulting fragmented single stranded DNA was then labeled with biotinylated ddUTP using the Enzo BioArray™ Terminal Labeling Kit (ENZO Life Sciences, Farmingdale, NY) following the manufacturers’ protocol. Affymetrix *E. Coli* Antisense GeneChip® arrays (Affymetrix, Santa Clara, CA) were handled at the University of Colorado DNA Microarray Facility according to manufacturer’s specifications using a GeneChip® Hybridization oven,GeneChip® Fluidics Station, GeneArray® scanner and GeneChip® Operating Software 1.1 (Affymetrix).

### Microarray Data Analysis

Data analysis was completed by utilizing SCALEs software developed by Lynch et al. according to author’s instructions [13]. Signal values corresponding to individual probe sets were extracted from the Affymetrix data file and partitioned into probe sets based on similar affinity values. Background signals for each probe were subtracted according to conventional Affymetrix algorithms (MAS 5.0). Non-specific noise was determined as the intercept of the robust regression of the difference of the perfect match and mismatch signal against the perfect match signal. Probe signals were then mapped to genomic position as the Tukey’s bi-weight of the nearest 25 probe signals and noise was removed by applying a medium filter with a 1000 bp window length. Gaps between probes were filled in by linear interpolation. This continuous signal was decomposed using an N-sieve based analysis and reconstructed on a minimum scale of 500 bp as described in further detail by Lynch *et al*. [13]. Signals were further normalized by the total repressor of primer (ROP) signal, which is on the library vector backbone and represents the signal corresponding to the total plasmid concentration added to the chip.

### Specific Growth and Killing Assays

For growth rate determination, each clone was inoculated from an −80°C stock, cultured in 5 ml LB with KAN and incubated overnight in a 15 ml conical tube at 37°C with shaking. Each overnight culture was diluted into MOPS minimal medium (containing KAN and 0.1% glucose) to an OD_600_ of 0.4 before and then sub-cultured, inoculating conical tubes with 1–10% (v/v) inoculum (starting OD_600_ = 0.1). Conical tubes (15 ml) were incubated at 37°C with shaking, and OD_600_ was monitored regularly. Triplicate vector control flasks were run in parallel for all growth experiments. For multiple clone experiments, a 96 well polypropylene plate was used and 100 µl samples were measured in triplicate every 30 minutes for 8 hours. Specific growth rate was calculated by determining the optimal fit of linear trend lines by analyzing the R^2^-value.

### Minimal Inhibitory Concentrations (MIC)

The MIC was determined aerobically in a 96 well-microtitre plate format as described previously [21]. Overnight cultures of strains were grown aerobically with shaking at 37°C in 5 ml LB medium (with antibiotic when required for plasmid maintenance). A 1% (v/v) inoculum was introduced into a 15 ml culture of MOPS minimal media. When samples reached mid-exponential phase, the culture was diluted to an OD_600_ of 0.5. The cells were further diluted 1∶1000 and a 90 µl aliquot was used to inoculate each well of a 96 well plate (∼10^5^ final CFU/ml). The plate was arranged to measure the growth of variable strains or growth conditions in increasing Bac8c concentrations, 0 to 60 µg/ml, in 2-fold increments [8]. MIC was determined as the lowest concentration at which no visible growth was observed after incubation at 37°C for 18 hr.

## Results and Discussion

### Selection for Bac8c Resistant Clones

We constructed plasmid-based genomic libraries of *E. coli* of defined insert size (∼500,000 clones) and employed the SCALEs method to identify *E. coli* genes for which overexpression improved growth in the presence of sub-lethal levels of Bac8c. We employed a decreasing gradient serial-transfer selection strategy, which we have shown previously improves both sensitivity (lack of false negatives) and selectivity (true positives) relative to alternative strategies (such as an increasing or flat concentration gradients) [13], [14], [15], [22]. We used three serial transfers with Bac8c concentrations ranging between the bactericidal and growth inhibitory levels (Fig. 1a). The SCALEs method allowed us to track each clone within the library population which enabled analysis of population dynamics to assess the strength and consistency of our selections. The enrichment for an increasingly fit population of clones demonstrates that selection occurred throughout the serial transfer studies (Fig. 1b). However, as was intended, only a moderate level of selective pressure was observed (as assessed by the moderate reduction in overall library diversity). Selections were designed this way so that we could identify a broad-range of genes associated with Bac8c resistance, as opposed to the use of a stronger selection pressure that only identifies the smaller set of genes that confer the highest levels of fitness. Fitness data for all genes for each selection can be found in Supplemental Table S2.

A genome-wide plot of all genes in the *E. coli* genome for which overexpression confers low-level Bac8c resistance is provided in Figure 1c. The fitness conferred by overexpression of each gene (at 125 bp resolution) is plotted around the genome for each serial transfer as defined in the legend. Thus circles i, ii, and iii correspond to each stage in the decreasing-gradient serial transfer selection. The SCALEs algorithm calculates a fitness score at 125 base pair resolution, which can easily be summarized across multiple 125 bp segments into fitness scores at the gene and/or multi-gene (i.e. operons or multi-gene library inserts) levels [see Lynch et al for detailed descriptions [13]]. The clones with the highest fitness values after the complete reverse gradient selection are indicated outside the circle.

It is interesting to note that the major differences in fitness among evaluated clones happened in the later stages of the selection, where the Bac8c concentration was reduced. This is consistent with our prior efforts where starting at a higher concentration selects first for the smaller set of clones that survive the initial shock and then allows for enrichment and separation of such true-positive clones by reducing the selective concentration. While not investigated here, in prior efforts we have shown how this approach improves enrichment for the most tolerant clones that might not grow as well at lower concentrations but are able to survive and/or grow at more selective concentrations. Our data suggests a similar pattern here, a relatively smooth fitness landscapes (inner two circles (i, ii) in figure 1c) after the first two selections followed by the emergence of a much rougher landscape in the final selection (the outer circle (iii) in figure 1c). Complete fitness data have been made available for additional analyses (see Table S1).

### Confirmation of Resistance

We picked twenty-one clones for further analysis that were identified as substantially enriched in the SCALEs analysis and obtained from plates of samples taken at the end of the enrichment studies. For each clone, we first confirmed an increase in resistance to Bac8c either through an increase in specific growth in the presence of the peptide or through an increase in minimum inhibitory concentration (MIC). We found that, in the presence of 3 µg/ml Bac8c, which caused an approximately 50% growth inhibition of the control, 9 of the 21 clones had a relative increase in growth rate compared to the control (*p-value<0.01) confirming the SCALEs predictions of Bac8c resistance (Figure 1D). Of the 21, three clones (*fusA-tufA, yicJ,* and *putA*) actually showed increased growth rate in the presence of the peptide relative to the absence of peptide.

We also tested each clone for an increase in MIC. Similar to the growth rate studies, 14 of the 21 clones demonstrated an increased MIC. These clones were identical with the exception of the *yajO* clone that only showed an increase in specific growth in the presence of the peptide, and the *pqiAB* clone that only showed an increase in MIC. Many of the clones that were not subsequently found to be resistant, showed a slower growth phenotype then the control, in the presence or absence of peptide, providing a possible explanation for their persistence despite lack of resistance (slow growth persistence phenotypes have been linked to antimicrobial resistance phenotypes in a number of studies) [23], [24].

### Further Confirmations and Cross-resistance

Cross-resistance studies were performed both to provide further confirmation of the relevance of the genes that when overexpressed led to Bac8c resistance, as well as to gain further insights into the potential modes of Bac8c action. We tested cross-resistance for seven of our Bac8c resistant clones towards the parental peptide of Bac8c, antimicrobials targeting membrane permeability, cell-wall synthesis, or protein synthesis, conditions triggering a general stress response, and agents influencing electron trafficking.

We first tested each of the clones against a panel of antibiotics that are known to: alter membrane permeability (polymyxin B and gramicidin), inhibit cell wall synthesis (vancomycin and carbenicillin), or inhibit protein synthesis (streptomycin) (Table 1). Most Bac8c tolerant clones exhibit no increase in tolerance to this collection of antibiotics. Only the *treB* clone was observed to demonstrate increased resistance to vancomycin and gramicidin. However, *treB* was not more resistant to carbenicillin (which also effects cell wall synthesis). These data suggest that collectively these clones conferred Bac8c resistance through relief of a mechanism of action separate from or more complex than the mechanisms employed by these antibiotics.

**Table 1:** The MIC for each compound was determined after 18 h incubation at 37°C.

Antibiotic	Mode of action	MIC (µg/ml)								
		Control	*appBC*	*lpd*	*treBC*	*yajO*	*putA*	*rpsLG-fusA*	*yicJ*	*dhaKLM*
Paraquat	electron donor	100	20	20	100	5	40	100	60	40
H_2_0_2_	hydroxyl radical	0.75	0.75	1	1	1	1	1	1	1
CCCP	uncoupler	25	50	50	N/A	50	50	N/A	N/A	50
Polymyxin B	pore formation	0.5	0.5	0.5	0.5	0.5	1	0.5	0.5	N/A
Vancomycin	cell membrane	<8	<8	<8	>16	<8	<8	<8	<8	N/A
Carbenicillin	cell wall synthesis	2	4	4	2	4	4	2	2	N/A
Gramicidin	pore formation	<8	<8	<8	32	<8	<8	<8	<8	N/A
Streptomycin	protein synthesis	64	64	64	64	64	64	32	64	N/A
Bac2A		8	8	8	64	8	8	64	8	N/A
Sub3		4	4	8	8	8	4	8	8	N/A
K24		4	8	8	8	8	4	8	4	N/A

Results are shown for several antimicrobials with Control (Empty vector), and the vector with the cloned genes *appBC* (alternative terminal oxidase), *lpd* (lipoamide dehydrogenase), *treBC* (trehalose PTS permease), *yajO* (putative NAD(P)H-dependent xylose reductase), *putA* (proline dehydrogenase), *rpsLG-fusA* (elongation factor EF-Tu), *yicJ* (galactose-pentose-hexuronide transporter family), and *dhaKLM* (dihydroxyacetone kinase).

We next wanted to know if the resistance to Bac8c extended to the parental peptide of Bac8c (Bac2A), and two additional peptides that were synthesized from Bac2A (K24, and Sub3) (Table 1). These peptides differ in net charge and length: K24 is a 9-mer with +3 charge (RVRWYRIFY-NH_2_), and Sub3 is a 12-mer with a +6 charge (RRWRIVVIRVRR-NH_2_). Several clones were cross-resistant to either or both Sub3 and K24. Clones *lpd, treB, yajO, rpsLG-fusA*, and *yicJ* were resistant to Sub3, and clones *appBC, lpd, treB, yajO,* and *rpsLG-fusA* were resistant to K24. Surprisingly, however, most of the clones that were resistant to Bac8c were not resistant to the parent peptide Bac2A. We found that only the *treB* and *rpsLG-fusA* clones exhibited cross-resistance to the parent peptide. These results further support the complexity of mechanism of action of antimicrobial peptides, wherein 1–2 amino acid changes in peptide sequence led to varying levels of susceptibility across a range of resistant clones. This data supports previous work done by Hilpert et. al. [8].

One possible explanation for how these clones confer resistance is that each elicits a cellular state that is generally more stress tolerance. To explore this possibility, we next tested resistance of the clones to heat shock. Only clones *yicJ*, and *rpsLG-fusA* demonstrated improved growth under heat shock conditions. Interestingly, of all clones tested these two clones were the only ones that had insert genes that are controlled by σ^24^, the heat shock sigma factor.

In previous studies, we showed that Bac8c interferes with electron trafficking [10], which involves a range of different electron acceptors and donors in *E. coli* such as NAD^+^/NADH and the various components of electron transport chain (as has been shown for other antibiotics previously [25]). We therefore assessed the cross-resistance of each of our clones to compounds with mechanisms of action specifically involving electron trafficking. Paraquat is a substrate that promotes electron relay, it accepts electrons from cellular reducing agents such as NADH, and transfers them to molecular oxygen. Both O_2_ and an electron source must be present for paraquat to elicit deleterious effects through generation of reactive oxygen species (ROS). It has also been shown that paraquat can inhibit NAD^+^ biosynthesis [26]. We found that all of our clones but *treBC* and *rpsLG-fusA* were consistently between 1.7 and 20 fold more susceptible to paraquat, which could be explained if they had either increased levels of NADH (or another relevant electron donor) or an increased level of baseline or induced ROS. To discriminate between these possibilities; we tested the sensitivity of these clones to H_2_0_2,_ an oxidizing agent, which should have the same effect as paraquat if these clones had elevated ROS. None of the clones were statistically more sensitive or resistant to H_2_0_2_. These results were consistent with the hypothesis that increased paraquat sensitivity was related to NAD^+^/NADH modulation rather than an increase in basal ROS levels, thus suggesting that most of our identified clones were conferring resistance through such modulation.

To further link any role of NAD^+^/NADH modulation in the resistance of our clones, several clones were tested for resistance to carbonyl cyanide m-chlorophenyl hydrazone (CCCP), an ionophore that disrupts the proton gradient by shuttling protons across the cytoplasmic membrane; this then uncouples electron transport chain based proton pumping from ATP synthesis. An increase in CCCP resistance has previously been correlated with an ability to uncouple substrate metabolism from oxidative phosphorylation [27]. We found that all tested clones were two-fold more resistant to CCCP, thus reinforcing the possible modulation of NAD^+^/NADH related electron trafficking in the various Bac8c resistant clones identified here.

### Conclusions

Our study used the SCALEs methodology to better understand the ability of the Bac8c antimicrobial peptide to avoid high-level resistance evolution in laboratory settings. Overexpression from the cloned gene as a method of resistance does not usually occur in the clinic; however it mimics both plasmid mediated resistance and regulatory mutations leading, for example, to increased expression of β-lactamases, efflux pumps, aminoglycoside modifying enzymes and LPS modifications [28]. Moreover, overexpression of specific genes leading to resistance can inform the mechanism of action in addition to prospective events and genes that may influence resistance. A selection process was designed to enrich for clones that were actively growing in the presence of Bac8c, with the expectation that such clones would out compete those that survived due to persistence or adaptive phenotypes. The selection gave us a diverse group of clones that had higher fitness in the presence of Bac8c and indeed were generally more resistant to this peptide. Our results underline our prior studies [10] in indicating that Bac8c likely targets multiple intracellular and membrane associated processes, and helped in clarifying some of these. We believe that the identification of potential resistance targets and mechanisms will provide some insight into how to design drugs that are not easily countered by the development of resistance.

It has often been observed that bacteria that become resistant to a specific antibiotic may develop new properties, including changes in susceptibility to other agents [29]. Our objective was to select for mechanisms of resistance to Bac8c through the use of SCALES, with the prospect that Bac8c is unlike other antibiotics. We were successful in selecting for resistance to Bac8c specifically as only one clone had tolerance to other agents. This work supports our previous publication that Bac8c has a very complex mechanism of action [10]. We hypothesize that Bac8c could be used synergistically with other antibiotics, antimicrobial peptides, or with antiseptics/disinfectants to decrease the required dose, and/or to prevent development of resistance. Of the Bac8c tolerant clones identified here, only one had a change in susceptibility to a diverse range of antibiotics. However, two antimicrobials that impact energy metabolism showed consistently altered MICs, with increased susceptibility to paraquat and resistance to CCCP identified for a range of clones. The former agent works through depletion of cellular reducing equivalents (i.e NADH, NADPH etc) and generation of ROS (which we propose was not relevant since no increased susceptibility to H_2_0_2_ was observed). Conversely bacteria that are resistant to uncouplers like CCCP either exclude these uncouplers or are still sensitive to the effects yet have altered properties that allow metabolism to proceed in the presence of a low membrane potential (ΔΨ) [27]. Together these results implicated modulation of electron trafficking in the resistance phenotypes of each of these clones.

We propose that clones fall into two resistance classes that are distinguished by their level of sensitivity to paraquat. Paraquat is a substrate that promotes electron relay and can be reduced by cellular NADPH and NADH. Both di-oxygen and electrons must be present for paraquat to elicit deleterious effects. Thus we speculate that there were two classes of Bac8c resistance: Class one (paraquat sensitive) included clones that were involved in processes that used NAD^+^/NADH, or NADP^+^/NADPH (Table 1), namely *appBC*, *lpd, yajO, putA, dhaKLM, motA,*and *flhCD*. The second class was not paraquat sensitive and might involve a more general stress response in resistance, namely *rpsLG-fusA* and *treB*. In that these speculations require much additional investigation, future studies focused on the mechanisms of how such a range of genetic strategies (i.e. overexpression of range of genes encoding diverse functions) might similarly lead to modulation of electron trafficking, and thus resistance, are of interest.

In conclusion, we sought here to connect two important concepts, namely that Bac8c disturbs energy metabolism when not fully disrupting the membrane, and that resistance to Bac8c does not develop with ease. These concepts were explored by a high-resolution, genome-scale selection and analysis utilizing the SCALEs method. The resultant clones supported a multimodal mechanism of action for Bac8c as suggested by our prior studies [10] as well as for other peptides, rather than suggesting that peptides are solely membrane active as has been suggested previously [30]. Our data connected the Bac8c disruption of energy metabolism with resistance mechanisms in *E.coli* likely involving energy metabolism related electron trafficking. Specifically, we observed that many of the genes conferring Bac8c resistance also conferred increased paraquat sensitivity, as well as CCCP resistance; thus reinforcing the mechanistic similarities underlying the resistance of each of these clone. Since each of these clones overexpresses a distinct genetic loci, this outcome serves to illustrate the relationships that exist between the complex modes of action and associated modes of resistance of this synthetic AMP.

## Supporting Information

Table S1 **Additional information for important clones.**
(DOCX)

Table S2 **Raw fitness data for Bac8c selections.**
(XLSX)
