# Supplementary material for: Genome-Wide Identification of Genes Conferring Energy Related Resistance to a Synthetic Antimicrobial Peptide (Bac8c)
Source: PLoS One. 2013 Jan 31;8(1):e55052. doi: 10.1371/journal.pone.0055052 (PMC3561415; doi:10.1371/journal.pone.0055052)
Supplement: Table S1 — Additional information for important clones. (DOCX) [file pone.0055052.s001.docx]

**Table S1.** Additional information for important clones.

| Clone | 654 rank | 654 fitness | COG |
| --- | --- | --- | --- |
| DhaKLM | 6 | 7.2 | G |
| Lpd | 14 | 6.86 | C |
| PutA | 50 | 6.4 | C |
| AppBC | 2467 | 3.86 | C |

^a^ Control MIC = 2 μg/ml
